# SOCIAL NETWORKS: BUILDING RESOURCES FOR DIVERSE AGING FAMILIES

**DOI:** 10.1093/geroni/igad104.0492

**Published:** 2023-12-21

**Authors:** Kristine Ajrouch, Toni Antonucci

## Abstract

Social networks provide tangible resources for older adults and their families. Yet, their formation and characteristics may vary across diverse aging contexts. Friedman and colleagues examine multiple dimensions of caregivers’ networks using a nationally representative sample (N=2,176). Latent profile analysis identified three classes of caregiving networks: (1) Large, sparse networks with relatively few kin and only some people who assist with caregiving tasks for care recipients; (2) Moderately-sized, somewhat dense, kin-based networks; (3) Small, very dense family networks where caregiving is shared across network members, all of which uniquely influence caregiver well-being. Hu and colleagues evaluate measurement invariance of objective and subjective social isolation between US-born and foreign-born older adults using a regionally representative sample (N=611). Findings show indicators of social network structure and perceived relationship quality may be useful for examining social isolation among older immigrants. Ajrouch and colleagues show distinct dimensions of networks differentially influence older adult’s cognitive function. Larger networks and higher proportion of family are positively related while higher contact frequency is negatively related to cognitive function. Using data from the Detroit-based Social Relations Study, Webster and Chin show the extent of similarity in health-related behaviors between older adults and their closest social network members. Analysis of 247 dyads illustrate associations between multiple social network factors (homophily, structure, and relationship quality) and similarity in these behaviors. Together, these papers advance theoretical and methodological approaches to the ways in which social networks serve as a resource across diverse aging contexts.

